# Clinical characteristics of Chinese pediatric patients positive for anti-NMDAR and MOG antibodies: a case series

**DOI:** 10.3389/fneur.2023.1279211

**Published:** 2024-01-05

**Authors:** Qingyun Kang, Hui Kang, Shulei Liu, Mei Feng, Zhen Zhou, Zhi Jiang, Liwen Wu

**Affiliations:** ^1^Department of Neurology, Hunan Children’s Hospital, Changsha, China; ^2^Department of Orthopaedics, General Hospital of Central Theater Command, Wuhan, Hubei, China

**Keywords:** children, MOG, NMDAR, anti-NMDAR encephalitis, overlapping autoimmune syndrome, clinical features

## Abstract

**Introduction:**

The cases of MOG-AD (MOG antibody-associated disorder) and anti-NMDAR encephalitis overlapping syndrome (MNOS) are rare, especially among pediatric patients, and their clinical understanding is limited. This study aimed to investigate the clinical manifestations, imaging findings, treatments, and prognosis of Chinese pediatric patients who tested positive for anti-NMDAR and MOG antibodies.

**Methods:**

This retrospective study enrolled 10 MNOS pediatric patients, 50 MOG-AD (anti-NMDAR antibody-negative), and 81 anti-NMDAR encephalitis (MOG antibody-negative) pediatric patients who were admitted from July 2016 to June 2022 and used their clinical data for comparison.

**Results:**

The MNOS patients had a significantly lower incidence of psycho-behavioral abnormalities and involuntary movements than anti-NMDAR antibody (+)/MOG antibody (−) patients and had a significantly higher incidence of sleep disorders, seizures, and psycho-behavioral abnormalities than MOG antibody (+)/anti-NMDAR antibody (−) patients. The MNOS patients had a significantly higher incidence of MRI abnormalities than the anti-NMDAR antibody (+)/MOG antibody (−) patients, while there was no significant difference in the incidence between the MNOS patients and the MOG antibody (+)/anti-NMDAR antibody (−) patients. No significant difference was seen in the initial mRS score between the three groups of patients. The anti-NMDAR antibody (+)/MOG antibody (−) patients had a higher rate of admission to the ICU, a longer length of in-hospital stay, and a higher rate of introduction to second-line treatment than the other two groups of patients. No significant difference was seen in the mRS score at the last follow-up and in the disease recurrence rate between the three groups. All these patients respond well to immunosuppressive therapy.

**Discussion:**

In the presence of psycho-behavioral abnormalities, sleep disorders, and frequent seizures in MOG-AD patients or demyelinating symptoms of the central nervous system or demyelinating lesions on head MRI in anti-NMDAR encephalitis patients, the coexistence of MOG and anti-NMDAR antibodies should be considered and would suggest a diagnosis of MNOS for these patients. Immunotherapy is effective among these patients and should be given possibly earlier.

## 1 Introduction

Anti-N-methyl-d-aspartate receptor (NMDAR) encephalitis is an autoimmune-mediated neuropsychiatric disorder caused by antibodies against the NR1 subunit of NMDAR, with seizures, psycho-behavioral abnormalities, motor disturbance, consciousness disturbance, speech disorder, memory loss, and autonomic dysfunction as the main clinical manifestations. Myelin oligodendrocyte glycoprotein (MOG) is part of normal myelin, the latter of which forms a protective sheath surrounding the nerve axon. MOG antibody (MOG-Ab) can cause damage to myelin and lead to inflammatory demyelinating disorders of the central nervous system (CNS), namely, MOG-Ab-associated disorder (MOG-AD). Increasing clinical evidence defines MOG-AD as an independent disease entity with a broad spectrum of clinical phenotypes including optic neuritis, meningoencephalitis, myelitis, brainstem encephalitis, and other special types, such as cranial neuritis, aseptic meningitis, and demyelinating pseudotumor (1–3). In recent years, mounting studies have shown that anti-NMDAR and MOG antibodies can be detected simultaneously or successively in the same patient, and their coexistence could lead to the overlapping or variation of clinical manifestations and simultaneous or sequential emergence of the clinical phenotypes of MOG-AD and anti-NMDAR encephalitis overlapping syndrome (MNOS) (4–7). MNOS cases among pediatric patients are rare, and their clinical understanding is limited as well. In this retrospective study, the clinical data of 10 MNOS pediatric patients positive for both anti-NMDAR and MOG antibodies were analyzed, and the pediatric patients positive for only anti-NMDAR or only MOG antibodies were enrolled for comparison to improve clinical understanding of MNOS and provide a reference for early diagnosis and appropriate treatment.

## 2 Materials and methods

### 2.1 Patients

The clinical data of 10 MNOS patients in the Department of Neurology, Hunan Children’s Hospital from July 2016 to June 2022 were retrospectively analyzed. In the same period, 81 patients with anti-NMDAR encephalitis and 28 patients with MOGAD were put into comparison. All patients met the diagnostic criteria for MOGAD (8) or anti-NMDAR encephalitis (9). The exclusion criteria included: (i) unclear diagnosis or incomplete data; (ii) presence of other specific autoantibodies against neurons or glial cells, except for NMDAR-Ab and MOG-Ab; (iii) coexistence of other autoimmune diseases; and (iv) secondary occurrence of MOGAD or anti-NMDAR encephalitis following viral encephalitis.

### 2.2 Clinical data

The clinical data of all patients collected for analysis included demographic characteristics, clinical manifestations, neurophysiological data, imaging and laboratory data, treatment, and prognosis. All patients were followed up for at least 6 months after discharge, with a median follow-up time of 33.5 (22.0, 52.6) months. The recurrence rate was evaluated at the conclusion of the follow-up period. The modified Rankin scale (mRS) was used to measure the degree of neurological disability at the time when a patient was in the worst condition (maximum score) and at the last follow-up (terminal score). An mRS score of ≤2 was defined as a good prognosis. New onset or deterioration of manifestations occurring at least 2 months after condition improvement or stabilization indicated a recurrence of anti-NMDAR encephalitis (9, 10). Relapses of MOG-AD were defined as the development of new neurological symptoms 1 month after onset of the initial episode or, in the case of phenotype of acute disseminated encephalomyelitis (ADEM), 3 months after onset of the initial episode (11). The clinical data of 10 pediatric patients positive for both MOG and anti-NMDAR antibodies (MNOS group) were retrospectively analyzed, and 81 anti-NMDAR antibody (+)/MOG antibody (−) pediatric patients (anti-NMDAR encephalitis group) and 50 MOG antibody (+)/anti-NMDAR antibody (−) pediatric patients (MOG-AD group) who visited the hospital during the same period were used as controls for comparison in clinical characteristics. Cell-based assays were used to detect anti-NMDAR and MOG antibodies.

### 2.3 Statistical analysis

All data were analyzed with IBM SPSS Statistics 26.0 software. The normally distributed measurement data were expressed as the mean ± standard deviation (SD), and their comparisons between groups were performed with analysis of variance. The skewed measurement data were expressed as the median and interquartile range (IQR), and their comparisons between groups were performed with the Kruskal–Wallis test. The enumeration data were expressed as number (n) and percentage (%), and their comparisons between groups were performed with the chi-square test or Fisher exact test. Pairwise comparisons between the three groups were adjusted for *p*-values using the Bonferroni method to retain the nominal alpha value (an adjusted *p*-value was equal to three times the original *p*-value). Correspondence analysis was used to study the correlation between the three groups for each clinical manifestation (categorical data). A two-sided *p*-value of <0.05 was considered statistically significant. In the study, correspondence analysis was conducted to analyze the correlation among the clinical manifestations of the three groups. An interactive summary table composed of categorical variables in the two-dimensional contingency table was used to reveal the corresponding relationship between different variables and which variables are the typical representatives of this category and to expose the relationship between variables and categories. The results were presented in the form of a scatter graph, and the spatial position of each scatter was used to objectively show the correlation among variables. The related attributes are located close together on the graph, and a closer distance represents a stronger correlation while a farther distance represents a weaker correlation.

## 3 Results

### 3.1 Demographic characteristics

This retrospective study enrolled 10 pediatric patients who tested positive for anti-NMDAR and MOG antibodies (MNOS group), 81 anti-NMDAR antibody (+)/MOG antibody (−) pediatric patients (anti-NMDAR encephalitis group), and 50 MOG antibody (+)/anti-NMDAR antibody (−) pediatric patients (MOG-AD group) for analysis and comparison. Table 1 shows the details of the demographic and clinical data of the 10 included MNOS pediatric patients. The results of the comparison of demographic and clinical characteristics between the anti-NMDAR encephalitis group, MOG-AD group, and MNOS group are shown in Table 2. There was no significant difference in the age of onset, the proportion of infants (≤3 years), and the sex ratio between the three groups (*p* > 0.05).

**Table 1:** Demographic and clinical characteristics of 10 pediatric patients with anti-myelin oligodendrocyte glycoprotein and anti-N-methyl-D-aspartate receptor antibody positive.

Serial number; sex	Age (month); onset time	The titer of anti-NMDAR antibody (serum/CSF)	Titer of MOG antibody (Serum/CSF)	Clinical manifestations	Brain MRI findings	CSF findings (WBC; protein)	Treatment	Max/terminal mRS scores
1; F	79; 1st	Negative/1:32	1:100/1:10	Fever, seizures, memory loss	T2/FLAIR hyperintense signal in the left hippocampus and internal capsule part of the basal ganglia; mild contrast enhancement	35 × 10^6^/L; 360 mg/L	IVIG + IVMP	3/0
2; F	67; 1st	Negative/1:3.2	1:100/Negative	Headache, vomiting, lethargy, irritability, diplopia, seizures, repetitive speech	T2/FLAIR hyperintense signal in bilateral frontal, parietal, occipital, and temporal parts, and the thalamus, T2 hyperintense signal in C2–C7 and T8–T12 spinal cord, and stripe-like T2 hyperintense signal in the right intra-orbital segment of the optic nerve	26 × 10^6^/L; 450 mg/L	IVIG + IVMP	4/0
3; F	96; 1st	1:320/1:1	1:100/1:10	Weak lower limbs, unsteady gait	T2/FLAIR hyperintense signal in parts of the bilateral frontal, parietal, and occipital cortex, and the corpus callosum and thalamus	10 × 10^6^/L; 460 mg/L	IVIG + IVMP	2/0
	101; 2nd	1:320/no	1:32/no	Psycho-behavioral abnormalities	Slightly longer T2 signal in the left occipital and frontal lobes	no	IVIG + IVMP	2/0
4; F	146; 1st	1:32/1:10	1:3200/1:100	Recurrent fever, somnolence, headache, joint pain, unsteady gait, slightly decreased vision	T2/FLAIR hyperintense signal in bilateral frontal, parietal, occipital, and temporal lobes, and cerebellum, T2 hyperintense signal in the T1–T5 spinal cord, and stripe-like T2 hyperintense signal in left optic nerve	70 × 10^6^/L; 330 mg/L	IVIG + IVMP	3/0
5; F	67; 1st	1:1000/1:10	1:1000/1:10	Poor spirit, unstable gait, drooping eyelids	Multiple patches of T2 hyperintense signal in the bilateral cerebellum, basal ganglia, and the white matter of bilateral frontal and parietal lobes	2 × 10^6^/L; 280 mg/L	IVIG + IVMP	3/0
6; M	149; 1st	Negative/1:32	1:100/Negative	Fever, headache, blurred vision, Psycho-behavioral abnormalities, poor sleep	Bilateral temporal, parietal, and occipital lobes, and left frontal and insular cortex were slightly swollen with patchy T2 hyperintense signal. Enhanced scanning showed stripe-like enhancement in some lesions and adjacent meninges.	130 × 10^6^/L; 410 mg/L	IVIG + Oral prednisone	3/0
7; F	150; 1st	1:10/1:1	1:100/1:10	Fever, lethargy, seizures, headaches	Patchy T2 hyperintense signal in bilateral basal ganglia and thalamus, and patchy and slight hyperintense signal in T2–T4 spinal cord.	38 × 10^6^/L; 240 mg/L	IVIG + IVMP	4/0
8; M	132; 1st	1:10/1:1	1:100/1:100	Seizures, delayed response, reduced speech	T2/FLAIR hyperintense signal in the right part of the cerebral cortex, curvilinear T2/FLAIR hypointense signal in the subcortical white matter near the right part of the cerebral cortex, and local T2 hyperintense signal in the right optic nerve.	28 × 10^6^/L; 370 mg/L	IVIG + IVMP	3/0
9; F	40; 1st	1:10/Negative	1:10/Negative	Fever, seizures, low spirit, irritability, slight memory loss	Extensively abnormal T2/FLAIR hyperintense signal in bilateral frontal, parietal, occipital, and insular lobes, bilateral basal ganglia, thalamus, brain stem, and bilateral cerebellar hemispheres.	181 × 10^6^/L; 660 mg/L	IVIG + IVMP	3/0
10; F	144; 1st	1:100/1:100	1:320/1:32	Headache, psycho-behavioral abnormalities, silence, involuntary movements, poor sleep, memory loss	Patchy T2/FLAIR hyperintense signal in the right side of the junction of the medulla oblongata and cervical spinal cord; slight contrast enhancement.	20 × 10^6^/L; 110 mg/L	IVIG + IVMP	3/0

NMDAR, N-methyl-d-aspartate receptor; MOG, myelin oligodendrocyte glycoprotein; MRI, magnetic resonance imaging; WBC, white blood cell; CSF, cerebrospinal fluid; Max/Min, maximum/minimum; F, female; M, male; FLAIR, fluid attenuation inversion recovery; IVIG, intravenous immunoglobulin; IVMP, intravenous methylprednisolone; mRS, modified Rankin scale.

**Table 2:** Comparison of demographic and clinical characteristics between the anti-NMDAR encephalitis group, MOG-AD group, and MNOS group.

Variables	Total	Anti-NMDAR encephalitis group (*n* = 81)	MOG-AD group (*n* = 50)	MNOS group (*n* = 10)	F/χ^2^ among the three groups	*p*-value among the three groups	*p*-value^*^ between the anti-NMDAR encephalitis group and the MOG-AD group	*p*-value^*^ between the anti-NMDAR encephalitis group and the MNOS group	*p*-value^*^ between the MOG-AD group and the MNOS group MOG
Age (mean ± SE, year)	6.77 ± 3.4	6.89 ± 3.58	6.15 ± 2.93	8.92 ± 3.48	2.937	0.056	0.682	0.220	0.057
Male (n, %)	58 (41.13%)	36 (44.44%)	20 (40.00%)	2 (20.00%)	2.238	0.327	>0.999	0.552	0.897
Infants (n, %)	14 (9.93%)	10 (12.35%)	4 (8.00%)	0 (0.00%)	#	0.599	>0.999	>0.999	>0.999
Headache / dizziness (n, %)	57 (40.43%)	28 (34.57%)	25 (50.00%)	4 (40.00%)	3.058	0.217	0.299	>0.999	>0.999
Consciousness disturbance (n, %)	71 (50.35%)	39 (48.15%)	27 (54.00%)	5 (50.00%)	0.424	0.809	>0.999	>0.999	>0.999
Fever (n, %)	59 (41.84%)	27 (33.33%)	27 (54.00%)	5 (50.00%)	5.720	0.057	0.084	0.940	>0.999
Seizures (n, %)	73 (51.77%)	57 (70.37%)	10 (20.00%)	6 (60.00%)	31.707	<0.001	<0.001	>0.999	0.049
Status epilepticus (n, %)	28 (19.86%)	23 (28.40%)	5 (10.00%)	0 (0.00%)	9.240	0.010	0.046	0.180	>0.999
Sensory disturbance (n, %)	15 (10.64%)	8 (9.88%)	6 (12.00%)	1 (10.00%)	0.151	0.927	>0.999	>0.999	>0.999
Visual disturbance (n, %)	27 (19.15%)	5 (6.17%)	19 (38.00%)	3 (30.00%)	21.046	<0.001	<0.001	0.122	>0.999
Speech disorder (n, %)	72 (51.06%)	56 (69.14%)	13 (26.00%)	3 (30.00%)	24.932	<0.001	<0.001	0.090	>0.999
Motor disturbance (n, %)	52 (37.14%)	33 (41.25%)	18 (36.00%)	1 (10.00%)	3.762	0.152	>0.999	0.248	0.443
Cognitive disorder/ memory loss (n, %)	64 (45.39%)	48 (59.26%)	11 (22.00%)	5 (50.00%)	17.407	<0.001	<0.001	>0.999	0.337
Ataxia (n, %)	28 (19.86%)	13 (16.05%)	13 (26.00%)	2 (20.00%)	1.924	0.382	0.546	>0.999	>0.999
Psycho-behavioral abnormalities (n, %)	75 (53.19%)	66 (81.48%)	4 (8.00%)	5 (50.00%)	67.090	<0.001	<0.001	0.114	0.013
Involuntary movements (n, %)	65 (46.10%)	62 (76.54%)	2 (4.00%)	1 (10.00%)	71.122	<0.001	<0.001	<0.001	>0.999
Sleep disorders (n, %)	54 (38.30%)	50 (61.73%)	1 (2.00%)	3 (30.00%)	46.987	<0.001	<0.001	0.261	0.038
Urinary retention (n, %)	13 (9.22%)	3 (3.70%)	10 (20.00%)	0 (0.00%)	#	0.007	0.014	>0.999	0.565
CSF pleocytosis (>5/mm^3^) (n, %)	107 (75.89%)	58 (71.60%)	40 (80.00%)	9 (90.00%)	2.362	0.307	0.925	0.843	>0.999
CSF proteinosis (n, %)	13 (9.22%)	3 (3.70%)	9 (18.00%)	1 (10.00%)	#	0.020	0.030	>0.999	>0.999
Abnormal EEG (slow waves) (n, %)	113 (80.14%)	67 (82.72%)	37(74.00%)	9 (90.00%)	2.133	0.344	0.808	>0.999	>0.999
Abnormal EEG (epileptic waves) (n, %)	35 (24.82%)	30 (37.04%)	3 (6.00%)	2 (20.00%)	16.093	<0.001	<0.001	>0.999	0.571
Abnormal head MRI (n, %)	70 (49.65%)	19 (23.46%)	41 (82.00%)	10 (100.00%)	53.303	<0.001	<0.001	<0.001	>0.999
Abnormal spinal MRI (n, %)	22 (36.67%)		18 (36.00%)	4 (40.00%)	#	>0.999			>0.999
Abnormal orbit MRI (n, %)	17 (28.33%)		14 (28.00%)	3 (30.00%)	#	>0.999			>0.999
IVIG treatment (n, %)	137 (97.16%)	81 (100.00%)	46 (92.00%)	10 (100.00%)	#	0.052	0.059	>0.999	>0.999
Methylprednisolone therapy (n, %)	139 (98.58%)	81 (100.00%)	48 (96.00%)	10 (100.00%)	#	0.261	0.432	>0.999	>0.999
Plasma exchange (n, %)	7 (4.96%)	6 (7.41%)	1 (2.00%)	0 (0.00%)	#	0.361	0.751	>0.999	>0.999
Second-line treatment (n, %)	20 (14.18%)	17 (20.99%)	3 (6.00%)	0 (0.00%)	7.484	0.024	0.072	0.595	>0.999
Poor prognosis (n, %)	9 (6.38%)	8 (9.88%)	1 (2.00%)	0 (0.00%)	/	0.221	0.456	>0.999	>0.999
Recurrence (n, %)	21 (14.89%)	11 (13.58%)	9 (18.00%)	1 (10.00%)	0.680	0.712	>0.999	>0.999	>0.999
Admission to the ICU (n, %)	18 (12.77%)	16 (19.75%)	2 (4.00%)	0 (0.00%)	8.464	0.015	0.049	0.598	>0.999
Maximum mRS score (mean ± SE)	3.52 ± 0.88	3.64 ± 0.93	3.4 ± 0.77	3.11 ± 0.87	5.359	0.069	0.362	0.137	0.777
Terminal mRS score (mean ± SE)	0.49 ± 1.01	0.59 ± 1.17	0.42 ± 0.75	0.11 ± 0.31	1.788	0.409	>0.999	0.781	0.544
Length of in-hospital stay (mean ± SE, day)	27.22 ± 18.42	33.09 ± 21.43	18.86 ± 7.54	19.14 ± 2.89	31.682	<0.001	<0.001	0.088	>0.999

#, no statistics using Fisher exact test; *, Bonferroni correction, adjusted *p*-value = original *p*-value × 3; MRI, magnetic resonance imaging; WBC, white blood cell; CSF, cerebrospinal fluid; ICU, intensive care unit; IVIG, intravenous immunoglobulin; mRS, modified Rankin scale; EEG, electroencephalogram.

### 3.2 Clinical characteristics

**Figure 1:**
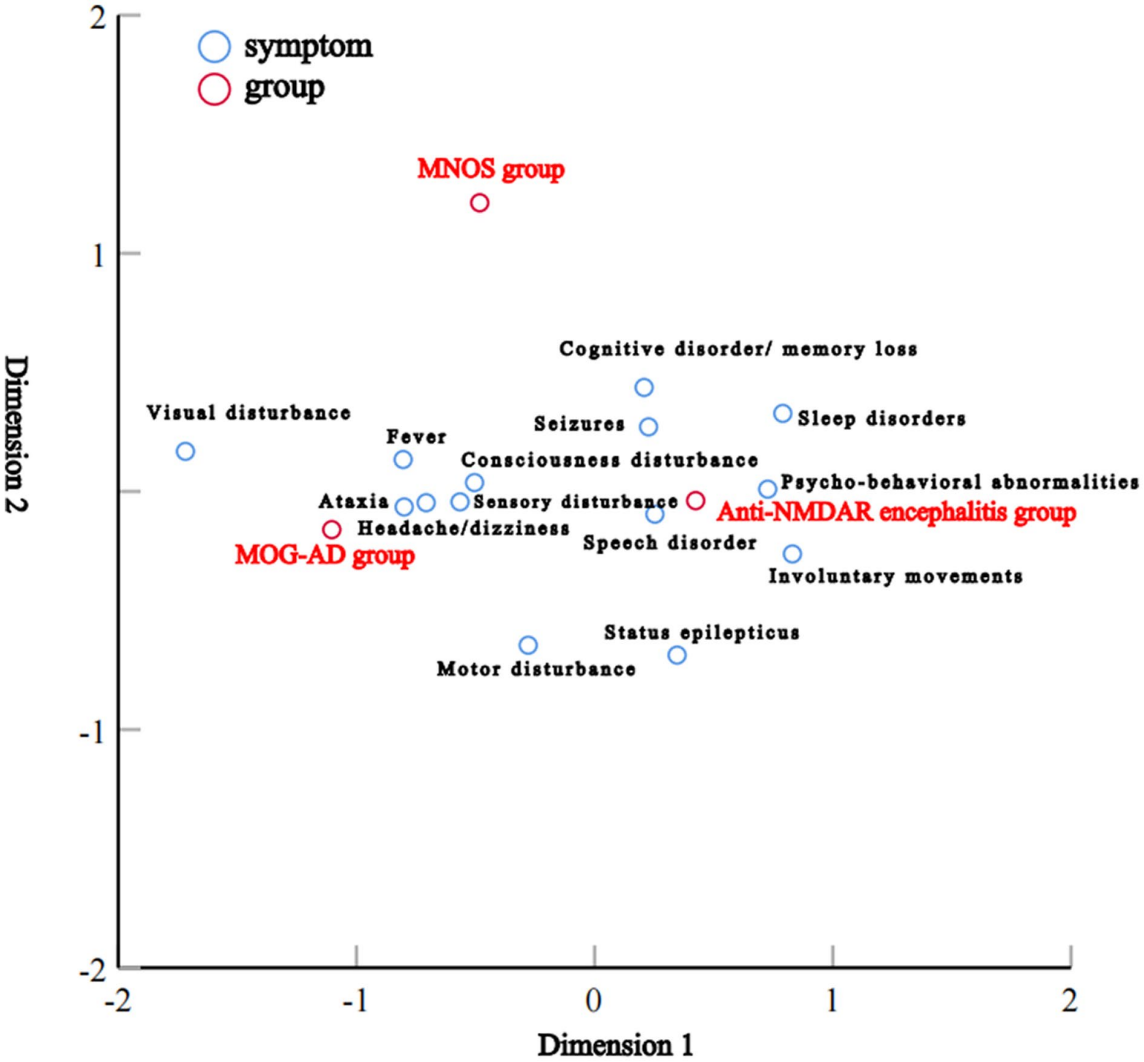
Results of correspondence analysis of clinical manifestations and groups.

**Figure 2:**
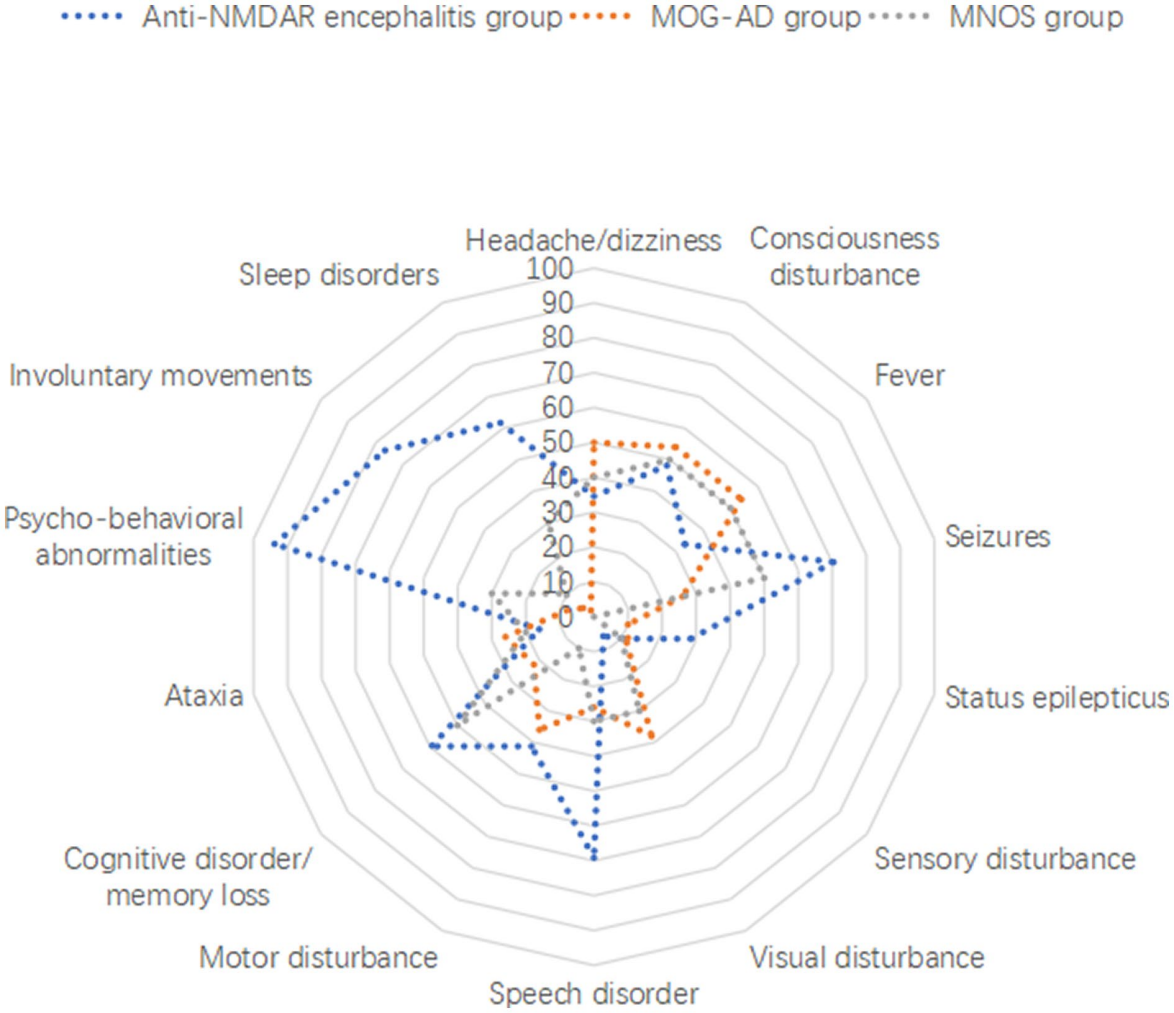
Distribution of clinical manifestations of anti-NMDAR encephalitis, MOG-AD, and MNOS patients.

Correspondence analysis was performed to investigate the association between the three groups and each clinical manifestation. The results showed that the MOG-AD group was closely associated with visual disturbance, ataxia, headache/dizziness, fever, and consciousness disturbance; the anti-NMDAR encephalitis group was closely associated with psycho-behavioral abnormalities, seizures, involuntary movements, sleep disorders, cognitive disorder/memory loss, consciousness disturbance, and speech disorder (Figure 1). The results of the distribution of clinical manifestations of the three groups showed that the clinical manifestations present in MNOS patients were also reported in the MOG-AD group or the anti-NMDAR encephalitis group; there was a difference in the incidence of clinical manifestations between MNOS patients and MOG-AD/anti-NMDAR encephalitis patients (Figure 2). Further comparisons between groups showed that the incidence of sleep disorders, seizures, and psycho-behavioral abnormalities was significantly higher in the MNOS group in comparison with the MOG-AD group (*p* < 0.05); the incidence of psycho-behavioral abnormalities and involuntary movements was significantly lower in the MNOS group in comparison with the anti-NMDAR encephalitis group (*p* < 0.05). Significant differences were seen in the incidence of seizures, status epilepticus, visual disturbance, speech disorder, cognitive disorder/memory loss, psycho-behavioral abnormalities, involuntary movements, sleep disorders, and urinary retention between the MOG-AD and anti-NMDAR encephalitis groups (*p* < 0.05).

### 3.3 Auxiliary examination

**Figure 3:**
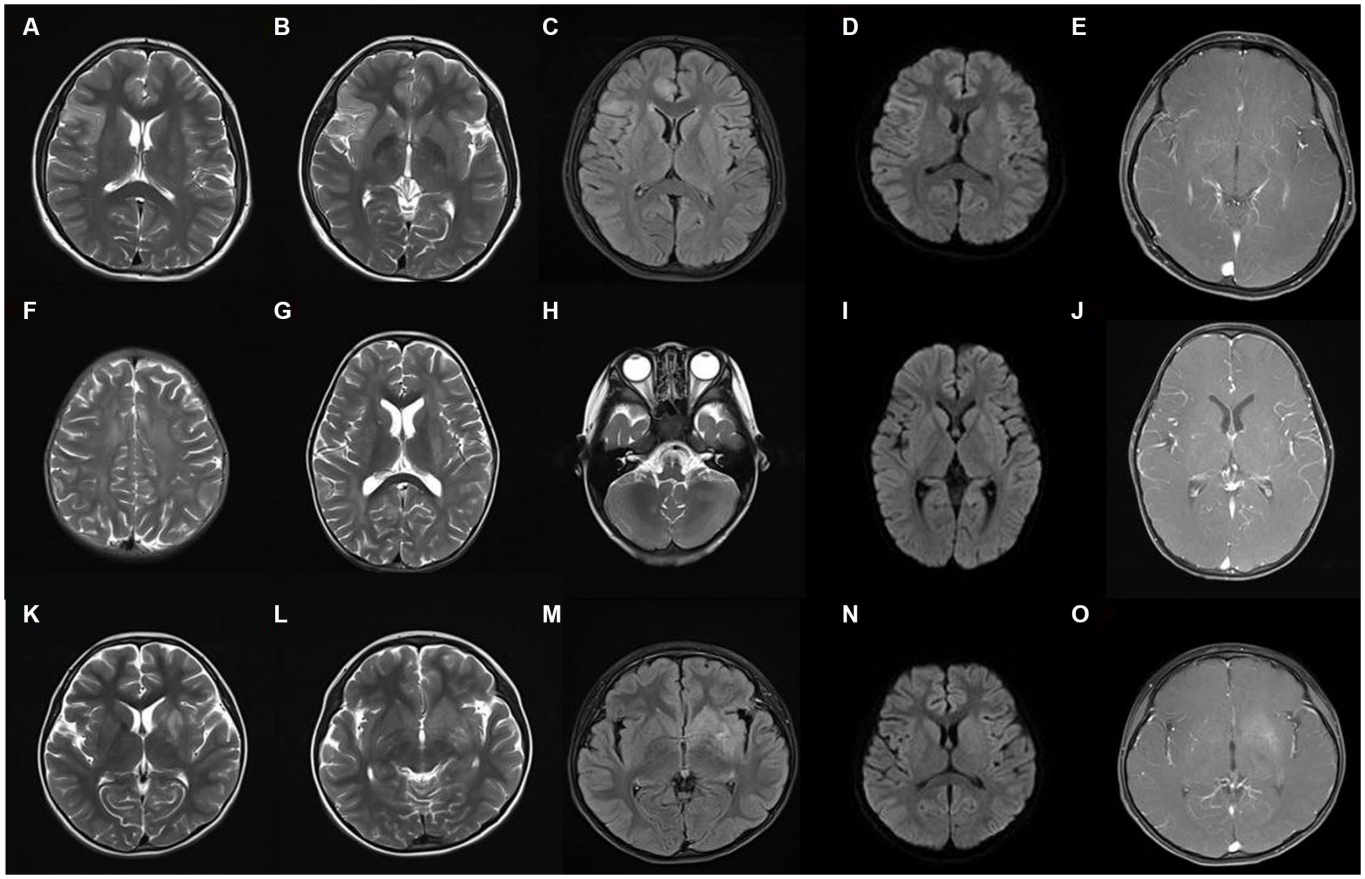
Typical cranial magnetic resonance imaging images of three groups of patients. **(A–E)** The images of a representative pediatric patient with intracranial lesions in the anti-NMDAR encephalitis group. The right frontotemporal insular lobe showed a partially and slightly swollen cortex, indistinct cortical-medullary boundary, patchy and slightly longer T1 signal, patchy and long T2 signal, and hyperintense on the FLAIR sequence, with no obvious diffusion limitation, and no abnormal enhancements on the enhanced scan; **(F–J)** The images of a representative pediatric patient in the MOG-AD group: the bilateral frontal, temporal, parietal, and occipital lobes, bilateral basal ganglia, and bilateral cerebellum showed multiple, patchy, slightly longer T1 and T2 signals and hyperintense on the FLAIR sequence, with no diffusion limitation, and multiple punctate enhancements on the enhanced scan; **(K–O)** The images of a representative pediatric patient in the MNOS group: the left hippocampus and the basal ganglia of the internal capsule showed patchy and slightly longer T1 signal, patchy and long T2 signal, and hyperintense on the FLAIR sequence, with blurred edge, no diffusion limitation, and slight enhancement on the enhanced scan.

The findings of cerebrospinal fluid tests and magnetic resonance imaging (MRI) in the three groups were analyzed (Table 2). No significant difference was seen in the results of routine cerebrospinal fluid tests between the three groups (*p* > 0.05). The proportion of abnormal cerebrospinal fluid protein cases in the MOG-AD group was significantly higher than that in the anti-NMDAR encephalitis group (*p* < 0.05). The typical cranial MRI images of patients in the three groups are displayed in Figure 3. In this study, we found that the rate of abnormal head MRI in the MOG-AD group was significantly higher than that in the anti-NMDAR encephalitis group (*p* < 0.05), and the rate in the MNOS group was significantly higher than that in the anti-NMDAR encephalitis group (*p* < 0.05). However, no significant difference was seen in the rate of abnormal head MRI between the MNOS group and the MOG-AD group (*p* > 0.05). Of the 141 pediatric patients in this study, only 3 patients in the anti-NMDAR encephalitis group had teratomas (including two ovarian teratomas and one pineal teratoma), and no tumors were found in the remaining patients.

### 3.4 Treatment and follow-up

The mRS scoring was performed for evaluating the patient’s condition, and the mRS score at the time when a patient was in the worst condition was selected as the maximum mRS score. As shown in Table 2, no significant difference was seen in the maximum mRS score between the three groups (*p* > 0.05). There were significant differences in the second-line treatment, admission to the intensive care unit (ICU), and length of in-hospital stay among the three groups (*p* < 0.05). All patients were followed up until December 2022. No significant difference was seen in the terminal mRS score obtained at the last follow-up between or among the three groups (*p* > 0.05). No significant difference was found in the rate of poor prognosis and the recurrence rate between or among the three groups (*p* > 0.05).

## 4 Discussion

The cases of antibody-mediated autoimmune diseases of the CNS in children have been increasingly reported with the widespread use of anti-neuronal antibody testing. The coexistence of multiple anti-neural antibodies is found clinically and refers to the presence of coexistent multiple autoantibodies in the same patient at the same time point or different autoantibodies in one patient at different time points. The presence of overlapping antibodies may arise from multiple immune responses elicited by a single antigen. Viral infections and other causes not only trigger inflammation and immune reactions within the CNS but also expose or modify neuronal proteins, which possess autoantigenic characteristics. In the abnormal immune environment of the CNS, autoimmune responses are initiated, leading to the generation of diverse autoantibodies. Regarding the phenomenon of antibody overlapping, the coexistence of NMDAR-Ab and MOG-Ab represents the most prevalent occurrence (12, 13). A previous study (14) found simultaneous or sequential demyelinating events in 23 (3.3%) of 691 anti-NMDAR encephalitis patients, and 9 (1.3%) of these patients tested positive for MOG antibodies. In recent years, with a deeper understanding of overlapping syndrome of CNS autoimmune diseases, medical practitioners routinely expand the detection to other anti-neural antibodies such as MOG antibodies among patients diagnosed with anti-NMDAR encephalitis. Previous studies reported 15 (16.85%) pediatric patients with anti-NMDAR encephalitis positive for MOG antibodies (15) and 5 (11.9%) MOG-AD patients positive for anti-NMDAR antibodies (16). Our study showed that approximately 11% of 91 anti-NMDAR antibody-positive patients were MOG antibody-positive and approximately 16.67% of 60 MOG antibody-positive patients were anti-NMDAR antibody-positive. Previous studies have shown that NMDAR-Ab and MOG-Ab can be present simultaneously or consecutively in MNOS patients. In our cohort, we identified the presence of both NMDAR-Ab and MOG-Ab simultaneously in 10 MNOS patients. The pathogenesis of MNOS is not clear. Functional NMDAR is reported to be present in oligodendrocytes (17); thus, in the pathogenic process of autoimmunity targeting oligodendrocytes, immune cells may mistakenly attack NMDAR and MOG autoantigens located in the same location, thereby producing anti-NMDAR and MOG antibodies, leading to the occurrence of the disease. In addition, the coexistence of MOG-Ab and GFAP-Ab or NMDAR-Ab and GFAP-Ab is also frequently observed, with some patients even exhibiting three or four overlapping antibodies. Multiple antibody positivity may also be associated with viral infections, disease development, and immune reconstitution during treatment (18–20).

In this study, the correspondence analysis suggested that consciousness disturbance, visual disturbance, ataxia, seizures, and speech disorder were the core manifestations of MOG-AD; psycho-behavioral abnormalities, seizures, involuntary movements, sleep disorders, cognitive disorder/memory loss, consciousness disturbance, and speech disorder were the core manifestations of anti-NMDAR encephalitis; MNOS patients had manifestations as presented in the MOG-AD group or anti-NMDAR encephalitis group, and consciousness disturbance, seizures, visual disturbance, psycho-behavioral abnormalities, and cognitive disorder/memory loss were common manifestations of MNOS. In comparison with the patients in the MOG-AD group, MNOS patients had a significantly higher incidence of sleep disorders, seizures, and psycho-behavioral abnormalities but a significantly lower incidence of urinary retention. This suggested that the incidence of urinary retention was decreased and the incidence of sleep disorders, seizures, and psycho-behavioral abnormalities was increased after the coexistence of anti-NMDAR and MOG antibodies, consistent with previous findings (21). Therefore, the presence of overlapping anti-NMDAR antibodies should be paid attention to when MOG-AD patients have manifestations such as psycho-behavioral abnormalities, sleep disorders, and frequent seizures. Compared with the anti-NMDAR encephalitis group, patients in the MNOS group had a significantly lower incidence of psycho-behavioral abnormalities and involuntary movements, a significantly higher incidence of demyelinating symptoms or signs such as visual disturbance, and a significantly higher rate of abnormal head MRI. This indicated that the phenotypes of anti-NMDAR encephalitis were atypical and easily complicated with CNS demyelinating manifestations after the coexistence of MOG and anti-NMDAR antibodies. Therefore, the possibility of overlapping MOG antibodies should be considered in clinical practice among such anti-NMDAR encephalitis patients.

Of the 141 included pediatric patients, only three patients in the anti-NMDAR encephalitis group had teratomas, and no tumors were found in the remaining patients, indicating a low proportion of concomitant tumors in these pediatric patients, especially young children, consistent with previous reports (12, 15). Even with a low incidence of tumors, the MOG-AD, anti-NMDAR encephalitis, and MNOS patients were prone to fever, vomiting, and headache/dizziness, suggesting that infectious factors may be the main cause of antibody-mediated CNS autoimmune diseases in children.

In this study, we found that the proportion of increased cerebrospinal fluid protein in the MOG-AD group was significantly higher than that in the anti-NMDAR encephalitis group (*p* < 0.05), suggesting that the inflammation was more pronounced in MOG-AD patients. In general, cranial MRI images in anti-NMDAR encephalitis patients are normal or mildly abnormal and show scattered punctate/patchy cortical/subcortical T2/FLAIR hyperintensities, and a few may have inflammatory demyelinating findings and involve the brainstem and white matter (22, 23). In our study, only 19 of 81 anti-NMDAR encephalitis patients (23.46%) had abnormal head MRI, which was lower than the previously reported (22), mainly because the cases with anti-NMDAR encephalitis secondary to viral encephalitis or multiple antibody positivity were excluded from our anti-NMDAR encephalitis group. In contrast, intracranial lesions are more common in MOG-AD patients and typically present as diffuse, fluffy, and poorly demarcated white matter lesions, which can be present in cortical gray matter, subcortical white matter, deep white matter, brainstem, basal ganglia, thalamus, and cerebellum, with contrast enhancement (17, 24–27). In the present study, 41 (82%) of 50 MOG-AD patients showed intracranial lesions on their head MRI images; all the 10 MNOS patients had abnormal head MRI which shared the characteristics of intracranial lesions in the MOG-AD patients, consistent with previous reports. This suggests that the possibility of MNOS needs to be considered when demyelinating changes in the brain such as multiple patchy or punctate foci of signal abnormalities are present on cranial MRI in anti-NMDAR antibody-positive patients.

In this study, all 141 patients were treated with immunotherapy. The first-line treatment involved IVIG treatment, glucocorticoid therapy, and plasma exchange. Second-line treatment, such as rituximab, cyclophosphamide, azathioprine, and mycophenolate mofetil, was given when the initial first-line treatment was not effective or if the patient relapsed. Approximately 21% of patients with anti-NMDAR encephalitis in our study did not respond well to first-line immunotherapy and required second-line immunosuppressive therapy. On the other hand, MOGAD patients responded well to first-line immunotherapy, and second-line immunotherapy was mostly used in MOGAD patients who experienced relapse (s). Our study revealed that the most commonly used second-line immunotherapeutic agent among patients with anti-NMDAR encephalitis or MOGAD was rituximab. However, there is limited high-quality research evidence supporting the efficacy of various second-line immunotherapeutic agents. MNOS patients had a similar response to first-line immunotherapy as the MOGAD patients. Our 10 MNOS patients were treated with a combination of steroids and IVIG, which could be considered the first-line treatment strategy for MNOS patients.

No significant difference was seen in the mRS score at the worst condition among the MOG-AD, anti-NMDAR encephalitis, and MNOS groups, but there was a significant difference in second-line treatment, admission to the ICU, and length of in-hospital stay among the three groups (*p* < 0.05). The anti-NMDAR encephalitis patients experienced a longer length of in-hospital stay, and more were admitted to the ICU and required second-line treatment, compared with the other two groups. No significant difference was found in the length of in-hospital stay and admission to the ICU between the MOG-AD and MNOS groups, suggesting that anti-NMDAR encephalitis was more severe than MOG-AD and MNOS. After immunotherapy, the symptoms/signs of patients in the three groups were significantly improved, and no significant difference was seen in the mRS score among the three groups. This suggests that patients in the three groups responded well to immunotherapy, and immunotherapy should be given possibly earlier after the diagnosis is confirmed.

Previous studies have indicated a high recurrence rate of MNOS, ranging from 46.2 to 63.4%, which is significantly greater than the recurrence rate observed in our MNOS patients. This difference may be attributed to the fact that our subjects are primarily children, whereas most of the previous studies have focused on adult patients. Additionally, the small sample size in our study may have introduced random errors.

In summary, the coexistence of anti-NMDAR and MOG antibodies can lead to the overlap of clinical phenotypes of anti-NMDAR encephalitis and MOG-AD. When MOG-AD is associated with atypical manifestations such as psycho-behavioral abnormalities, sleep disorders, and frequent seizures, the presence of anti-NMDAR antibodies should be considered. When anti-NMDAR encephalitis patients have CNS demyelinating manifestations or demyelinating changes such as multiple patchy or punctate foci of signal abnormalities on head MRI, the presence of MOG antibodies should be considered. It is recommended that these patients should be tested for NMDAR and MOG antibodies at the same time. Such patients respond well to immunosuppressive therapy and immunotherapy should be given possibly earlier. This retrospective study may have selection bias due to the small sample size of MNOS patients; therefore, it is necessary to further expand the sample size and perform prospective multicenter large-scale studies in future to further clarify the clinical characteristics of patients positive for MOG and anti-NMDAR antibodies, facilitate early definitive diagnosis, and find better treatment strategies.

## Data availability statement

The datasets presented in this article are not readily available because of ethical and privacy restrictions. Requests to access the datasets should be directed to the corresponding author.

## Ethics statement

The studies involving humans were approved by the Ethics Committee of Hunan Children’s Hospital. The studies were conducted in accordance with the local legislation and institutional requirements. Written informed consent for participation in this study was provided by the participants’ legal guardians/next of kin. Written informed consent was obtained from the individual(s) for the publication of any potentially identifiable images or data included in this article.

## Author contributions

QK: Writing – original draft, Data curation, Methodology, Validation. HK: Methodology, Writing – review & editing. SL: Data curation, Writing – review & editing. MF: Data curation, Writing – review & editing. ZZ: Methodology, Writing – review & editing. ZJ: Methodology, Writing – review & editing. LW: Supervision, Writing – review & editing.
